# Supplementary figures and images for: Immunohistochemical and Ultrastructural Analysis of Adult Neurogenesis Involving Glial and Non-Glial Progenitors in the Cerebellum of Juvenile Chum Salmon Oncorhynchus keta
Source: Int J Mol Sci. 2025 Sep 23;26(19):9267. doi: 10.3390/ijms26199267 (PMC12524278; doi:10.3390/ijms26199267)

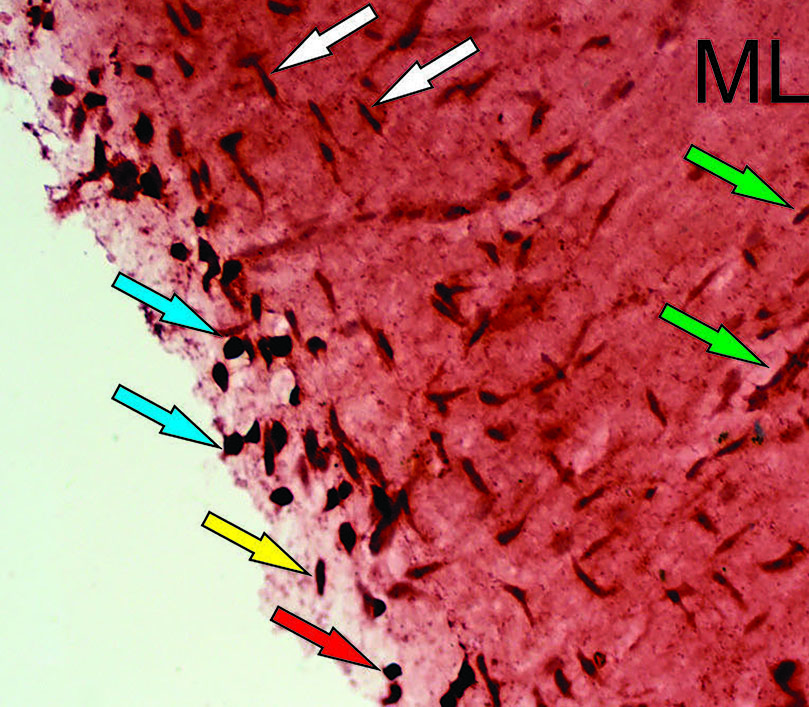

Supplement: Supplementary file 1 [file ijms-26-09267-s001.zip › Figure S1A.jpg]

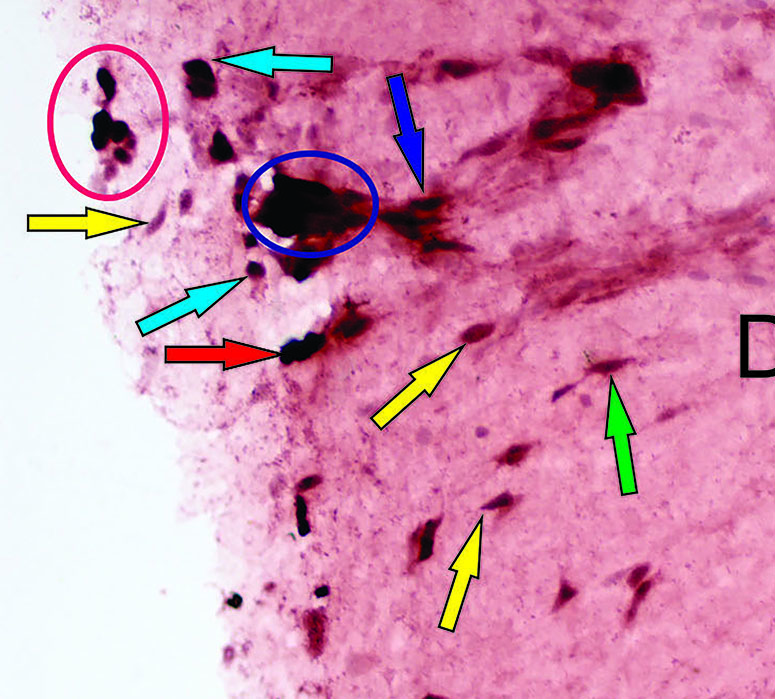

Supplement: Supplementary file 1 [file ijms-26-09267-s001.zip › Figure S1B.jpg]

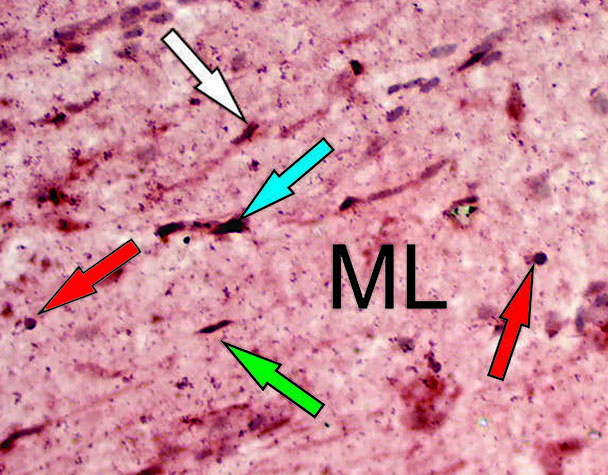

Supplement: Supplementary file 1 [file ijms-26-09267-s001.zip › Figure S1C.jpg]

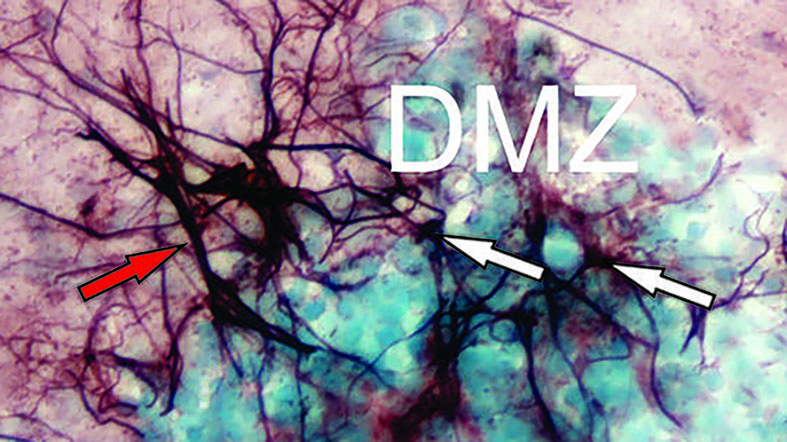

Supplement: Supplementary file 1 [file ijms-26-09267-s001.zip › Figure S2A.jpg]

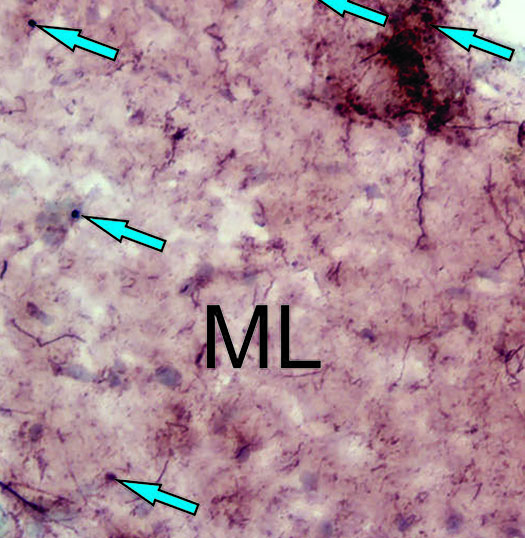

Supplement: Supplementary file 1 [file ijms-26-09267-s001.zip › Figure S2B.jpg]

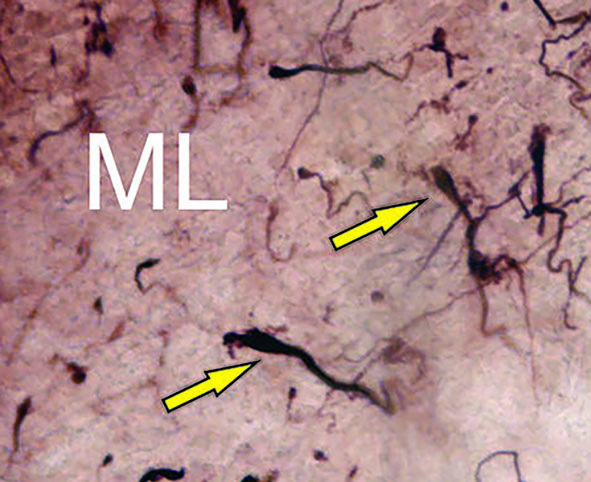

Supplement: Supplementary file 1 [file ijms-26-09267-s001.zip › Figure S2C.jpg]

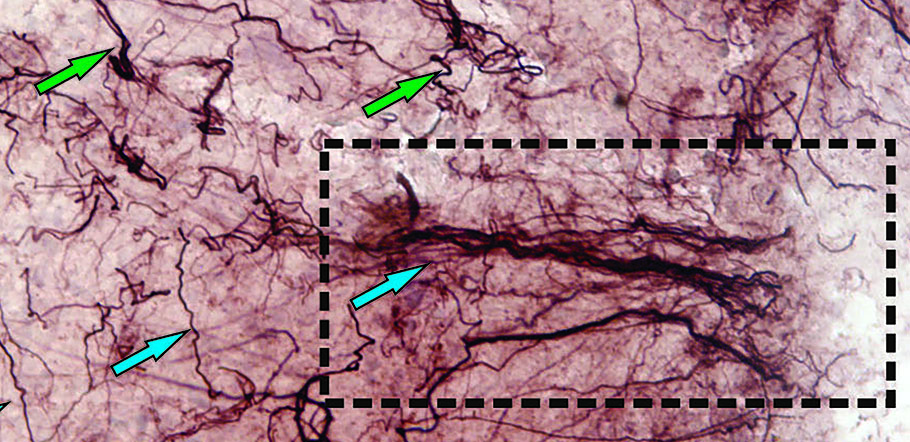

Supplement: Supplementary file 1 [file ijms-26-09267-s001.zip › Figure S2D.jpg]

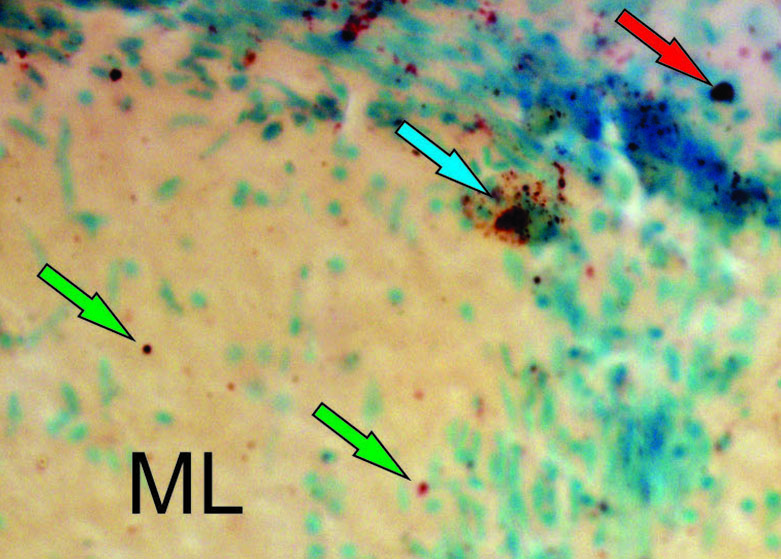

Supplement: Supplementary file 1 [file ijms-26-09267-s001.zip › Figure S3A.jpg]

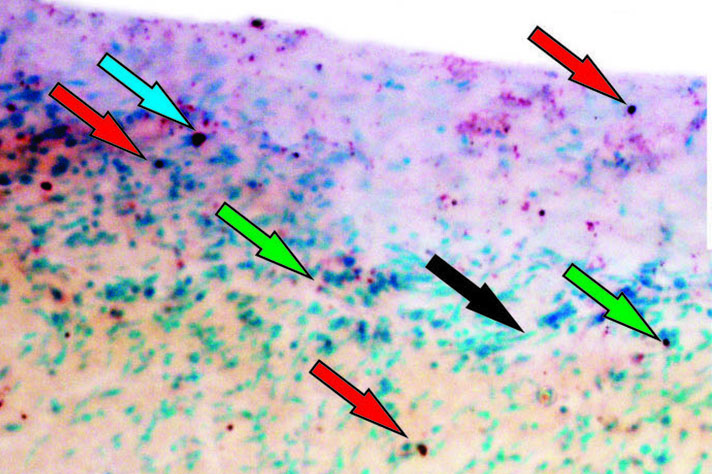

Supplement: Supplementary file 1 [file ijms-26-09267-s001.zip › Figure S3B.jpg]

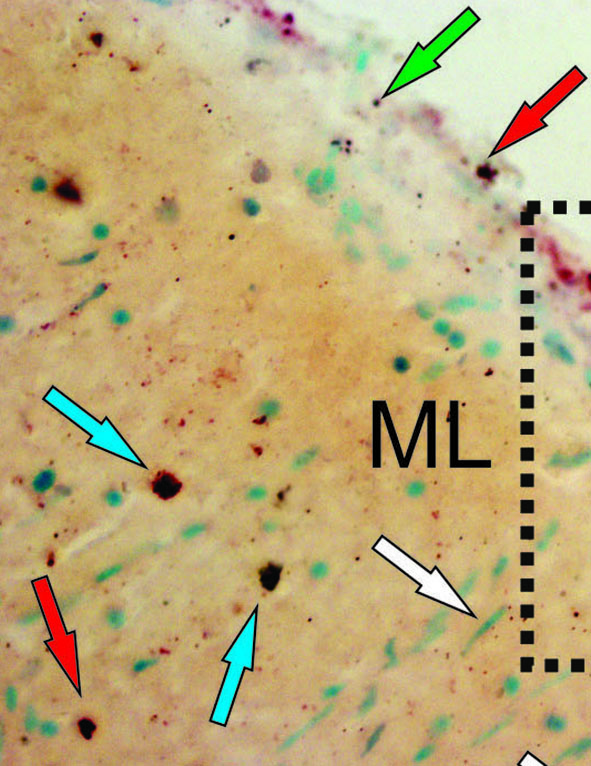

Supplement: Supplementary file 1 [file ijms-26-09267-s001.zip › Figure S3C.jpg]

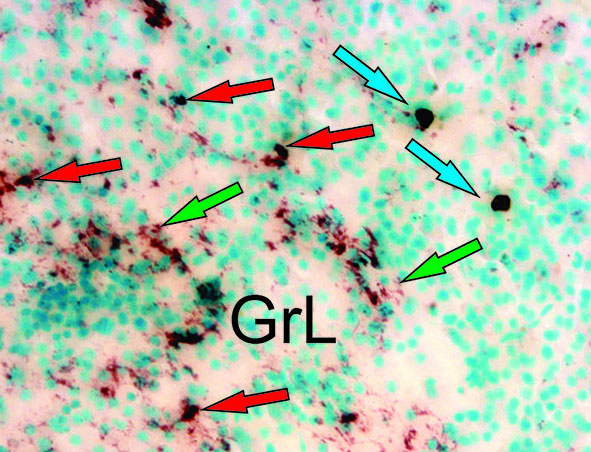

Supplement: Supplementary file 1 [file ijms-26-09267-s001.zip › Figure S3D.jpg]

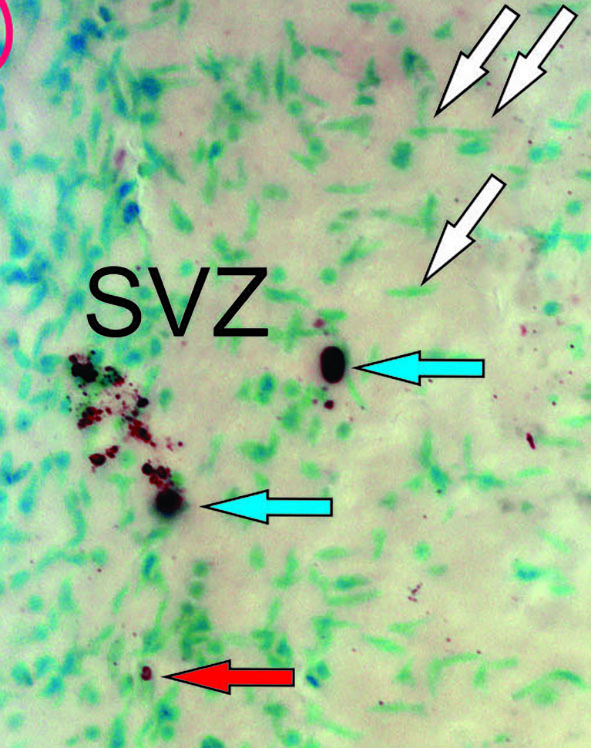

Supplement: Supplementary file 1 [file ijms-26-09267-s001.zip › Figure S3E.jpg]

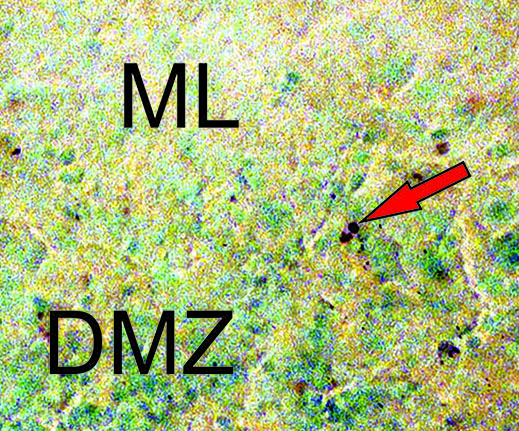

Supplement: Supplementary file 1 [file ijms-26-09267-s001.zip › Figure S4R.jpg]

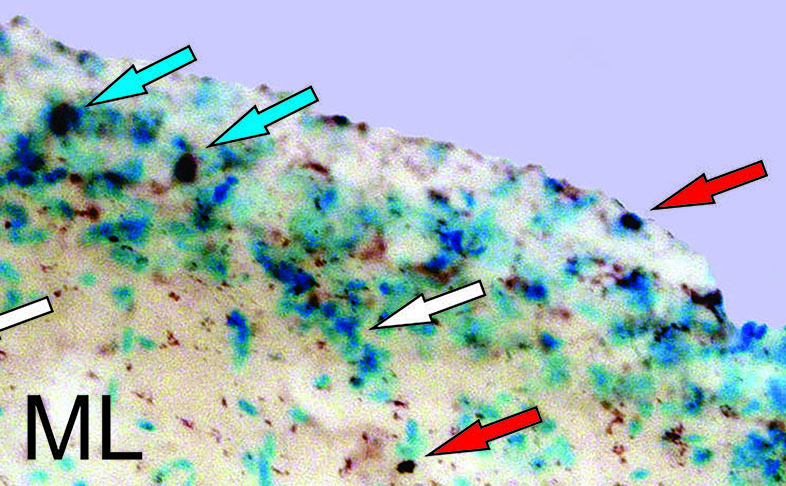

Supplement: Supplementary file 1 [file ijms-26-09267-s001.zip › Figure S4S.jpg]

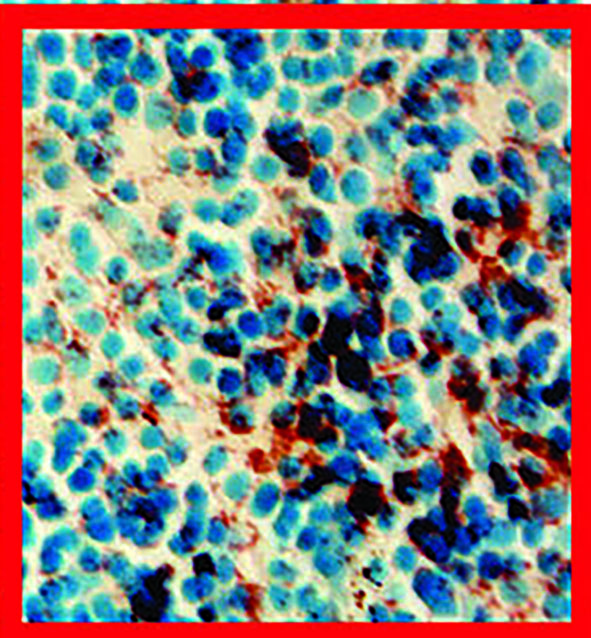

Supplement: Supplementary file 1 [file ijms-26-09267-s001.zip › Figure S4T.jpg]

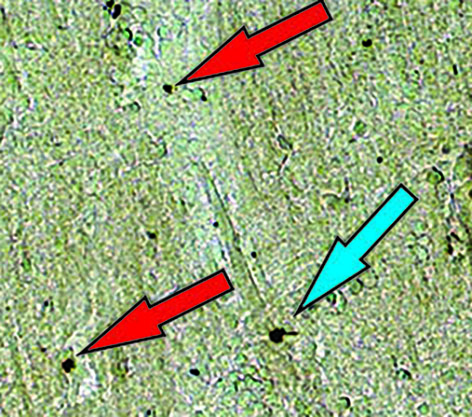

Supplement: Supplementary file 1 [file ijms-26-09267-s001.zip › Figure S4U.jpg]

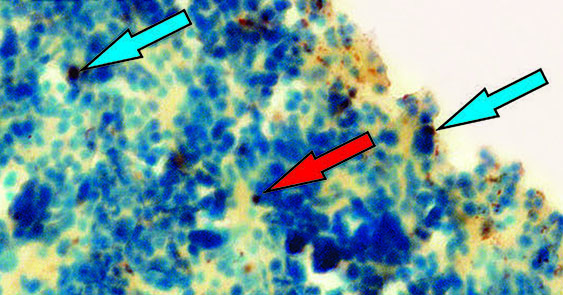

Supplement: Supplementary file 1 [file ijms-26-09267-s001.zip › Figure S4V.jpg]

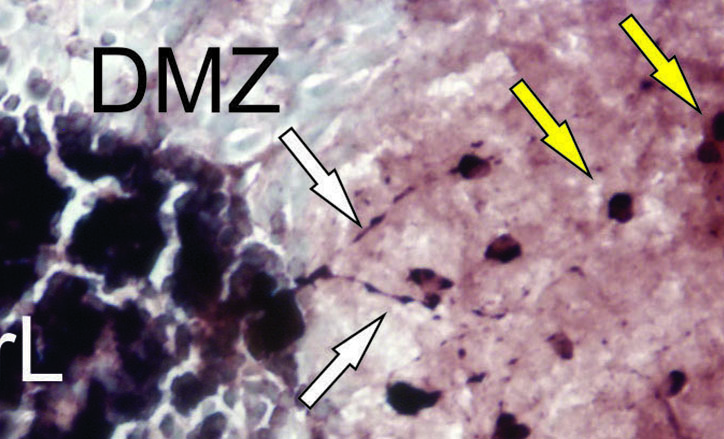

Supplement: Supplementary file 1 [file ijms-26-09267-s001.zip › Figure S5S.jpg]

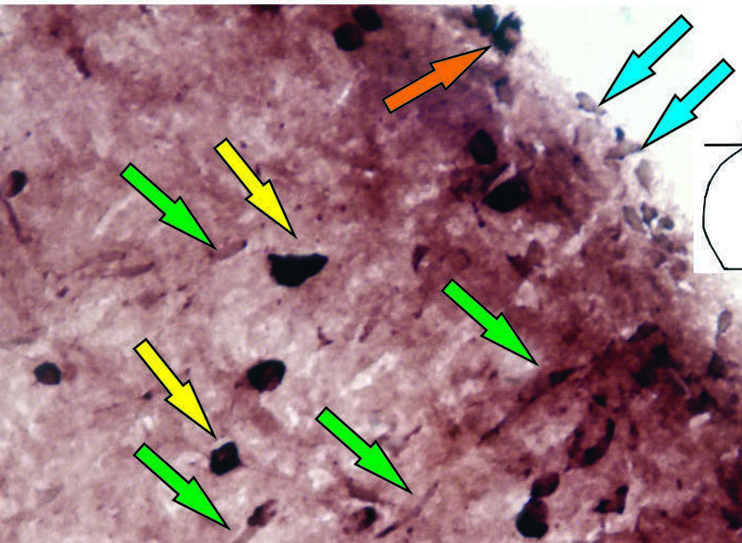

Supplement: Supplementary file 1 [file ijms-26-09267-s001.zip › Figure S5T.jpg]

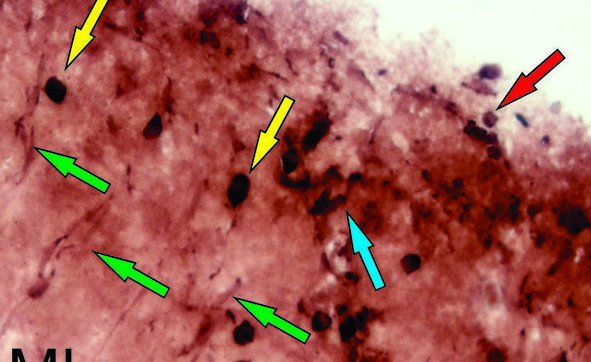

Supplement: Supplementary file 1 [file ijms-26-09267-s001.zip › Figure S5U.jpg]

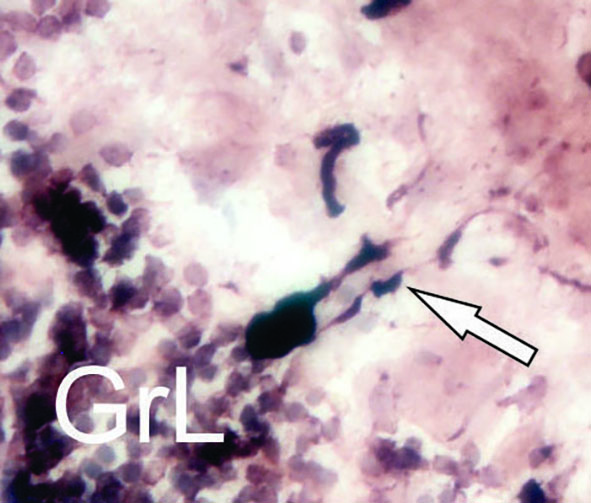

Supplement: Supplementary file 1 [file ijms-26-09267-s001.zip › Figure S5V.jpg]

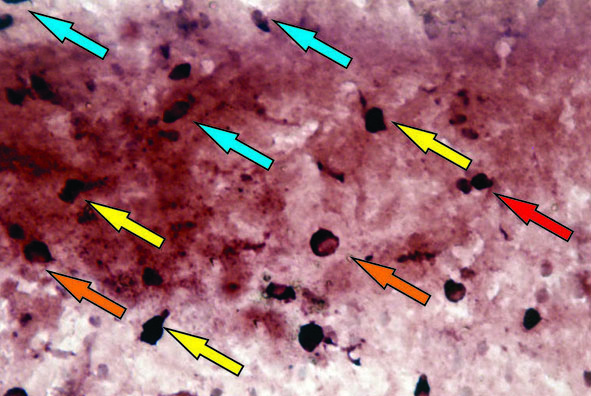

Supplement: Supplementary file 1 [file ijms-26-09267-s001.zip › Figure S5W.jpg]

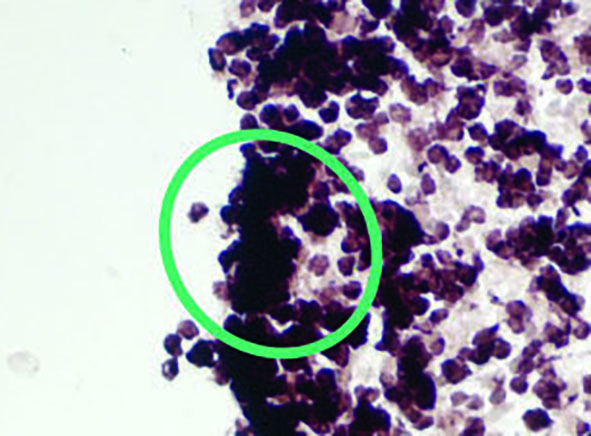

Supplement: Supplementary file 1 [file ijms-26-09267-s001.zip › Figure S5X.jpg]

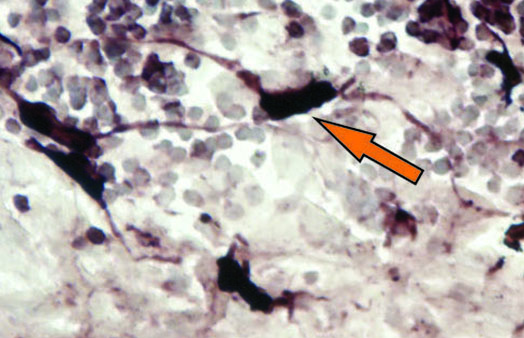

Supplement: Supplementary file 1 [file ijms-26-09267-s001.zip › Figure S5Y.jpg]
